# A Different Approach to the Treatment of Tracheal Stenosis

**DOI:** 10.7759/cureus.47496

**Published:** 2023-10-22

**Authors:** Deny Sung, Urooj Zahid, Mandeep Singh

**Affiliations:** 1 Internal Medicine, St. Joseph’s Medical Center, Stockton, USA; 2 Pulmonary and Critical Care Medicine, St. Joseph’s Medical Center, Stockton, USA

**Keywords:** recurrent tracheal stenosis, noncompliant endovascular catheter, tracheostomy tube, balloon dilatation, tracheal stenosis

## Abstract

Tracheal stenosis can occur from several malignant and non-malignant conditions, including vascular ring/aneurysms, tracheomalacia, bronchomalacia, endotracheal tubes (ETT), tracheostomy tubes, mucus plug, burn, trauma, tuberculosis, etc. Significant tracheal stenosis typically requires management by bronchoscopy, dilatation, or surgical resection. Bronchoscopic balloon dilatation is a simple, inexpensive, safe, and minimally invasive method that can be used to dilate airway stenosis and restore adequate airflow. A Montgomery tracheostomy tube is often used as a combined tracheal stent and airway device. We describe a 68-year-old female with tracheal stenosis, which was managed with an unconventional approach, a non-compliant endovascular balloon, due to resource scarcity.

## Introduction

Tracheal stenosis can occur from several malignant and non-malignant conditions, including vascular ring/aneurysms, tracheomalacia, bronchomalacia, endotracheal tubes (ETT), tracheostomy tubes, mucus plug, burn, trauma, tuberculosis, etc. Prolonged intubation is one of the most common causes, and the mechanism is thought to be due to decreased capillary perfusion pressure from the ETT cuff pressure, leading to ischemia and tissue damage [1,2]. Significant tracheal stenosis typically requires management by bronchoscopy, dilatation, or surgical resection. Bronchoscopic balloon dilatation is a simple, inexpensive, safe, and minimally invasive method that can be used to dilate airway stenosis and restore adequate airflow [2-5]. Generally, the balloon is inflated to pressures between 3 and 9 atmospheric pressures (ATM) to achieve a dilatation of 8 to 20 mm in diameter [6]. A Montgomery tracheostomy tube is often used as a combined tracheal stent and airway device. The Montgomery T-tube provides a stable airway for at least six months [7].

## Case presentation

**Figure 1:**
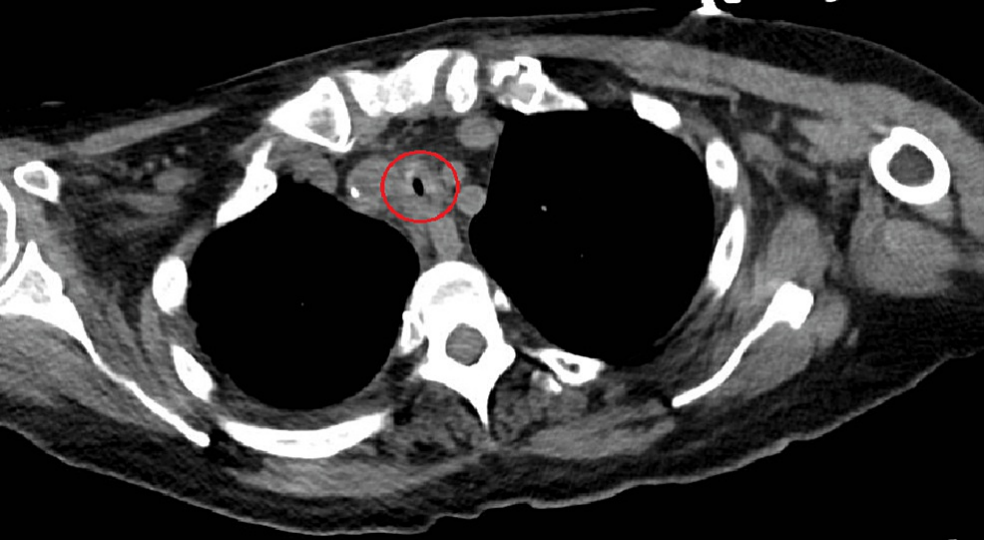
CT chest in axial view showing tracheal stenosis

**Figure 2:**
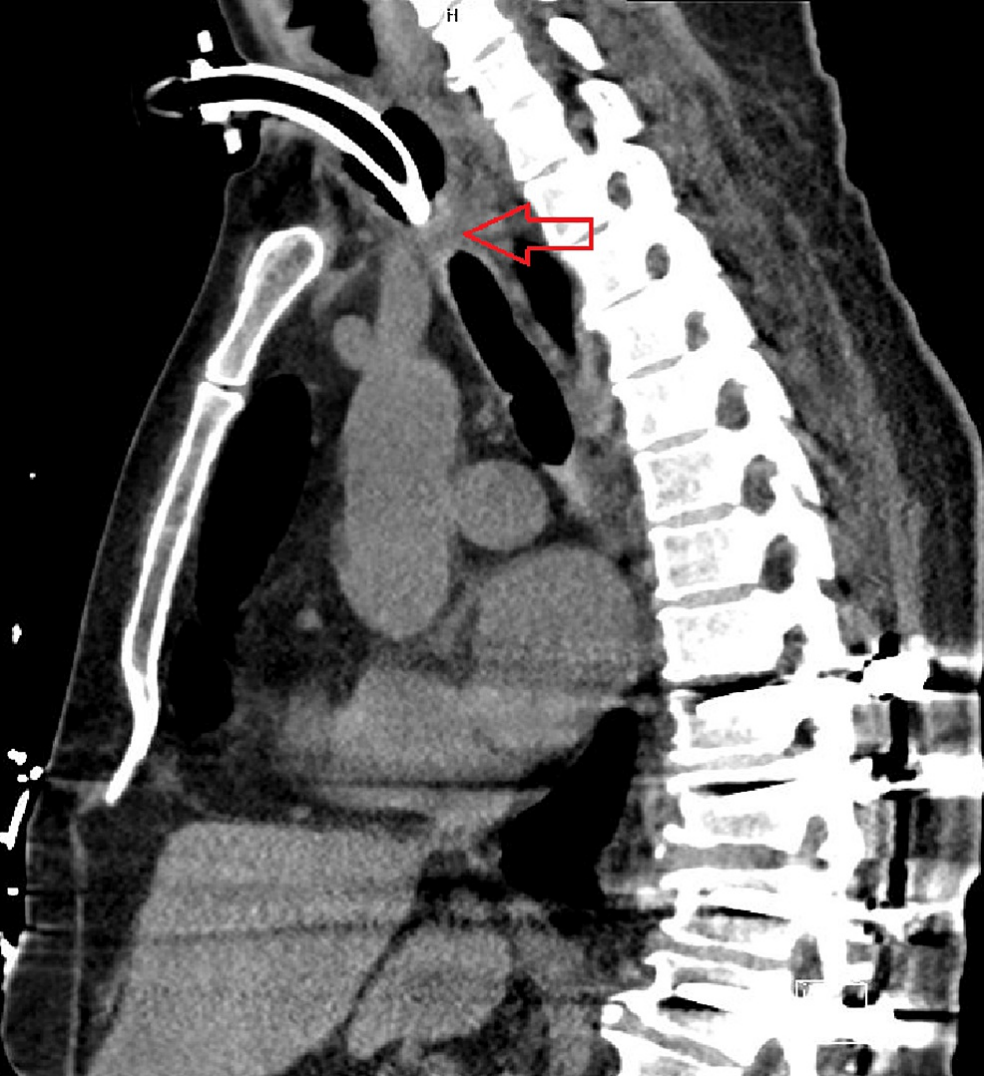
CT chest in sagittal view showing tracheal stenosis distal to tracheostomy tube

The patient is a 68-year-old female with a past medical history of subdural hematoma from a motor vehicle accident, tracheostomy and gastrostomy tube dependence, and seizure disorder who was found to have low tidal volumes (Vt) at her skilled nursing facility. Bronchoscopy revealed a subglottic tracheal stenosis immediately distal to the level of the tracheostomy tube as the cause of the low Vt (Figures 1-2).

The tracheostomy tube was replaced with a smaller, 5 mm endotracheal tube to secure the airway. The patient was then transferred to a tertiary care facility for balloon dilatation since this procedure is not available in our facilities. A month later, the patient was transferred back to our facilities after balloon dilatation, stenting, and the replacement of the stent with a Montgomery tracheostomy tube.

During the re-admission, she had an episode of cardiopulmonary arrest from thick secretions and mucous plugging of the tracheostomy tube. ROSC was achieved, the Montgomery tube was removed, and a 6.5-mm endotracheal tube was inserted through the tracheostoma. A 6.5-mm XLT distal-cuffed tracheostomy tube was eventually safely switched with the ETT. A week later, the patient was transferred out and sent back the next day after a second tracheal dilation and Montgomery tube reinsertion.

A couple of months later, the patient developed respiratory distress, and tracheal stenosis was found again distal to the caudal arm of the Montgomery tube. The patient's trachea was found to have a 3.5-mm tracheal stenosis, which was too small for endotracheal tube insertion. Without a secure and patent airway, the patient was not able to be transferred to a tertiary center for repeat tracheal dilation. Thus, cardiothoracic (CT) surgery was consulted to assist in securing a reliable airway for this critical patient. The CT surgeon used a 10 mm balloon followed by a 12 mm noncompliant balloon at 20 ATM in order to dilate the stenotic area. After dilatation of the tracheal stenosis and placement of a Shiley size 6 XLT tracheostomy tube, the patient was weaned off the ventilator and discharged to a skilled nursing facility with a tracheostomy collar.

## Discussion

A previous study showed that the rates of immediate success of dilatation and symptom improvement with balloon dilatations were greater than 90%, although some patients required multiple sessions [2]. Initially, our patient was able to be transferred to a tertiary care facility where the appropriate tools were available. However, during her third need for dilatation, she did not have a secure airway, and an intervention was needed in-house. The cardiothoracic surgeon used a non-compliant endovascular balloon on our patient. Although 10 mm non-compliant balloon catheters are generally used in endovascular procedures, due to a lack of resources and the balloon’s ability to endure high pressures to dilate calcified vessels [8], it was used on our patient for a highly resistant stenotic trachea. The patient also had previously tolerated an 11-mm external-diameter tracheostomy tube; thus, it was collectively decided that a 10-mm noncompliant balloon would be safe to use and not rupture the trachea. The ATM required to dilate our patient’s stenosis was two to three times higher than the ATM used in traditional pulmonary balloon dilation catheters [2,4,6]. We suspect that the endovascular balloon’s higher atmospheric pressure capacity may have helped in opening our patient’s highly resistant stenotic trachea.

## Conclusions

Further research and exploration of different dilatation devices may be warranted to expand current treatment options for tracheal stenosis, as seen in our patient, whose tracheal stenosis was successfully expanded with an endovascular balloon and higher balloon pressures. This would be beneficial in areas where resources and specialty facilities are scarce.
